# Heterologous Production of β-Caryophyllene and Evaluation of Its Activity against Plant Pathogenic Fungi

**DOI:** 10.3390/microorganisms9010168

**Published:** 2021-01-14

**Authors:** Fabienne Hilgers, Samer S. Habash, Anita Loeschcke, Yannic Sebastian Ackermann, Stefan Neumann, Achim Heck, Oliver Klaus, Jennifer Hage-Hülsmann, Florian M. W. Grundler, Karl-Erich Jaeger, A. Sylvia S. Schleker, Thomas Drepper

**Affiliations:** 1 Institute of Molecular Enzyme Technology, Heinrich-Heine-University Düsseldorf, Forschungszentrum Jülich, Wilhelm-Johnen-Straße, 52428 Jülich, Germany; f.hilgers@fz-juelich.de (F.H.); a.loeschcke@fz-juelich.de (A.L.); y.ackermann@fz-juelich.de (Y.S.A.); o.klaus@fz-juelich.de (O.K.); j.hage-huelsmann@fz-juelich.de (J.H.-H.); karl-erich.jaeger@fz-juelich.de (K.-E.J.); 2 INRES—Molecular Phytomedicine, University of Bonn, Karlrobert-Kreiten-Str. 13, 53115 Bonn, Germany; samer@uni-bonn.de (S.S.H.); sneuman1@uni-bonn.de (S.N.); grundler@uni-bonn.de (F.M.W.G.); 3 Institute of Bio- and Geosciences (IBG-1: Biotechnology) Forschungszentrum Jülich, Wilhelm-Johnen-Straße, 52428 Jülich, Germany; a.heck@fz-juelich.de

**Keywords:** terpenoids, sesquiterpene production, *Rhodobacter capsulatus*, β-caryophyllene, bioactivity, phytopathogens, plant pathogenic fungi, plant growth-promoting bacteria

## Abstract

Terpenoids constitute one of the largest and most diverse groups within the class of secondary metabolites, comprising over 80,000 compounds. They not only exhibit important functions in plant physiology but also have commercial potential in the biotechnological, pharmaceutical, and agricultural sectors due to their promising properties, including various bioactivities against pathogens, inflammations, and cancer. In this work, we therefore aimed to implement the plant sesquiterpenoid pathway leading to β-caryophyllene in the heterologous host *Rhodobacter capsulatus* and achieved a maximum production of 139 ± 31 mg L^−1^ culture. As this sesquiterpene offers various beneficial anti-phytopathogenic activities, we evaluated the bioactivity of β-caryophyllene and its oxygenated derivative β-caryophyllene oxide against different phytopathogenic fungi. Here, both compounds significantly inhibited the growth of *Sclerotinia sclerotiorum* and *Fusarium oxysporum* by up to 40%, while growth of *Alternaria brassicicola* was only slightly affected, and *Phoma lingam* and *Rhizoctonia solani* were unaffected. At the same time, the compounds showed a promising low inhibitory profile for a variety of plant growth-promoting bacteria at suitable compound concentrations. Our observations thus give a first indication that β-caryophyllene and β-caryophyllene oxide are promising natural agents, which might be applicable for the management of certain plant pathogenic fungi in agricultural crop production.

## 1. Introduction

Among secondary metabolites, terpenoids including the class of sesquiterpenoids represent one of the largest and most diverse groups with over 80,000 known compounds, mostly isolated from plants [1,2,3,4]. Based on their number of carbon atoms, they can be divided into the subclasses of hemi- (C_5_), mono- (C_10_), sesqui- (C_15_), di- (C_20_), tri- (C_30_), tetra- (C_40_) and polyterpenes (>C_40_) [5,6]. In general, the terpenoid synthesis starts from the two isoprene intermediates isopentenyl pyrophosphate (IPP) and dimethylallyl pyrophosphate (DMAPP), which are provided either by the mevalonate (MVA) pathway or by the 1-deoxy-d-xylulose 5-phosphate (DXP) pathway, also known as the 2-*C*-methyl-d-erythritol 4-phosphate (MEP) pathway. While the MVA pathway uses acetyl-Coenzyme A (acetyl-CoA) as a substrate and is predominantly found in eukaryotes (e.g., mammals, plants, and fungi), archaea and a few bacteria [7], the DXP pathway starts from glyceraldehyde-3-phosphate (GAP) and pyruvate and primarily occurs in bacteria, cyanobacteria, and green algae [8]. Starting from IPP and DMAPP, the elongation of linear prenyl pyrophosphates is catalyzed by prenyltransferases via head-to-tail condensations and results in C_10_ geranyl pyrophosphate (GPP), C_15_ farnesyl pyrophosphate (FPP), and C_20_ geranylgeranyl pyrophosphate (GGPP). Finally, GPP is used as a precursor molecule for the synthesis of monoterpenoids, FPP for sesqui- and triterpenoid production, and GGPP for di- and tetraterpenoid biosynthesis. Terpenes exhibit manifold functions in plant physiology and development, including photoprotection (carotenoids), communication (e.g., pinene), or repellant activity against predators and parasites (e.g., verbenone, β-caryophyllene) [9,10,11]. Furthermore, terpenes are of commercial interest for the pharmaceutical sector due to their various bioactivities suitable for the treatment of pathogen infections, inflammation, or cancer [12,13]. For example, the sesquiterpene farnesol shows inhibitory effects against antibiotic-resistant *Staphylococci*, not only inhibiting the growth of planktonic cells in free suspension but also suppressing biofilm formation of *Staphylococcus aureus*, *Staphylococcus epidermidis*, and *Burkholderia pseudomallei* [14,15,16,17]. In the past, these compounds were exclusively obtained from essential oils of natural plant sources, requiring complex and time-consuming downstream processing. β-caryophyllene, for example, was extracted from *Cannabis sativa* [18], clove basil, *Ocimum gratissimum* [19], or representatives of the plant genus *Cordia*, such as *Cordia verbenaceae* [20]. However, the application of microorganisms as heterologous hosts allows the establishment of alternative, cost-effective, and sustainable biotechnological production processes [21,22,23,24,25,26]. As the efficiency of such processes strongly depends on the achieved production titers, metabolic engineering of the applied hosts together with the optimization of the respective secondary metabolite pathways has gained more attention in the recent past [1,27,28,29,30,31]. So far, terpenoids were mostly produced in the heterologous hosts *Escherichia coli* and *Saccharomyces cerevisiae* [32,33,34,35,36]. However, in recent studies, the terpene production in less common microbes such as phototrophs has also been established and optimized, as for example documented by the *Rhodobacter*-based production of β-farnesene, nootkatone, valencene, and amorphadiene [23,37,38,39,40], or the production of various terpenes in cyanobacteria [41].

The phototrophic non-sulfur α-proteobacteria of the genus *Rhodobacter* feature some unique physiological properties, making them interesting microbial hosts for heterologous terpene production: (i) the cell membrane is commonly considered to be a critical determinant in terpenoid production since it can function as a storage compartment for the involved enzymes and metabolites [42,43]. In this context, *Rhodobacter* seems to be particularly suited for terpene production since the bacterium can form an extended intracytoplasmic membrane system (ICM), thereby providing a naturally enlarged reservoir for membrane-bound enzymes and terpenes [44,45]. (ii) As these phototrophic bacteria produce the carotenoids spheroidene and spheroidenone using the DXP pathway [46,47], they further offer a robust and effective isoprenoid metabolism that can be engineered for efficient terpenoid production. (iii) *Rhodobacter* species enable photo(hetero)tropic growth in low-cost minimal media at relatively high growth rates, allowing the utilization of sunlight as an energy source for sustainable cultivation and production processes. Recent studies could demonstrate that engineering the isoprenoid precursor biosynthesis can lead to a strong increase of sesqui- and triterpenoid formation in *R. capsulatus* [39,48,49] and *R. sphaeroides* [38,40,50,51,52]. In particular, the co-expression of a terpene synthase with the FPP synthase IspA, and/or enzymes constituting the heterologous MVA pathway, resulted in enhanced production of the corresponding plant terpenoids.

A major problem in agricultural crop production is the large number of plant-damaging animals such as insects, mites, and nematodes or pathogens including viruses, bacteria, and fungi, some of which lead to high economic losses of around 60% globally [53]. One of the most widely distributed and destructive pathogens of plants causing white mold disease in more than 400 host plants all over the world is the fungus *Sclerotinia sclerotiorum* (Lib.) de Bary [54]. Another devastating example of fungal diseases is the plant vascular wilt caused by the *Fusarium* species [55]. Other fungal pathogens such as *Phoma lingam* [56,57], *Alternaria brassicicola* [58], and *Rhizoctonia solani* [59,60] also cause major yield reduction in important crops. To control these pathogens and due to the rapidly growing world population and the resulting increase of food consumption, farmers are using synthetic and biological substances as fertilizers, pesticides, or growth regulators side by side with the cultivation of resistant or tolerant plant varieties [61,62,63,64]. Each of these methods has its limitations, but so far, the use of pesticides is the most convenient and commonly used method. Nevertheless, these can have numerous severe side effects on the environment, including the soil [65]. The soil is inhabited by an enormous diversity of organisms that are important players in maintaining a functional ecosystem and that comprise microorganisms with beneficial properties for plant development and health. For that reason, effective and sustainable alternatives are needed. Firstly, plants, as a part of a complex ecosystem, can produce enormous amounts of secondary metabolites for their survival and maintenance. Phenolics and terpenes are examples of metabolites that are produced by plants and act as antimicrobial agents and feeding deterrents [66,67,68,69,70,71,72]. The presence of a wide range of terpenes encouraged their use as nature-inspired plant protection agents in agriculture or their use for drug development. One of the commonly stress-associated terpenes is the sesquiterpene β-caryophyllene [73,74,75]. As mentioned in the previous section, various studies showed that β-caryophyllene exhibits diverse biological activities against many organisms. From a plant protection perspective β-caryophyllene was reported to promote plant growth, to induce plant defense genes, to attract entomopathogenic nematodes, and to be active against certain plant pathogenic bacteria and fungi [76,77,78,79,80].

In this study, we therefore aimed to use the modular co-expression of DXP/MVA genes in combination with the strictly controlled P*_nif_* promoter to reconstitute the pathway of the plant sesquiterpene β-caryophyllene in *R. capsulatus* and to optimize the production under phototrophic growth conditions. For heterologous sesquiterpene production, the β-caryophyllene synthase QHS1 from *Artemisia annua* was used. Since this terpene offers a variety of beneficial bioactivities, we further evaluated the potential use of β-caryophyllene and its oxygenated derivative β-caryophyllene oxide as nature-derived fungicides. To this end, the bioactivity of β-caryophyllene/oxide against both representative plant growth-promoting bacteria and phytopathogenic fungi was investigated.

## 2. Materials and Methods

### 2.1. Bacterial Strains and Cultivation Conditions

The *Escherichia coli* strain DH5α and strain S17-1 were used for cloning and conjugation of plasmid DNA [81,82]. *E. coli* cells were cultivated at 37 °C using LB agar plates or liquid medium (Luria/Miller, Carl Roth^®^, Karlsruhe, Germany), containing kanamycin (25 µg mL^−1^) when appropriate. *R. capsulatus* SB1003 [83] and SB1003-MVA [39], encompassing the chromosomally located genes *mvaA*, *idi*, *hsc*, *mvk*, *pmk* and *mvd* (also designated as MVA gene cluster) from *Paracoccus zeaxanthinifaciens*, were used for plant terpene production. All *R. capsulatus* strains used in this study were either cultivated on PY agar plates [84] containing 2% (*w*/*v*) Select Agar (Thermo Fisher Scientific, Waltham, MA, USA) or in RCV liquid medium [85] at 30 °C. Both media were supplemented with rifampicin (25 µg mL^−1^). For cultivation of the recombinant *Rhodobacter* strain SB1003-MVA, gentamicin (4 μg mL^−1^) was further added to the medium. If not stated otherwise, photoheterotrophic cultivation was conducted under anaerobic conditions and permanent illumination with bulb light (2500 lx), as described previously [39]. All bacterial strains and plasmids used in this study are listed in Table S1 (Supplementary Materials). All strains for bioactivity and minimum inhibitory concentration (MIC) evaluation are listed in the respective results section.

### 2.2. Construction of Expression Vectors

The expression vectors used in this study are based on the pRhon5Hi-2 vector carrying the promoter of the *nifH* gene for heterologous gene expression [39]. The sequence of β-caryophyllene synthase QHS1 from *A. annua* (UniProt: Q8SA63) was used to generate an appropriate synthetic gene whose DNA sequence is suitable for the codon-usage of *R. capsulatus*. For DNA sequence adaptation, the Codon Optimization Tool by IDT Integrated DNA Technologies and the Graphical Codon Usage Analyzer tool were used [86]. The 1.7-kb *QHS1* gene was obtained from Eurofins Genomics. The synthetic DNA fragment was flanked by appropriate restriction endonuclease recognition sequences (*Xba*I/*Hin*dIII). The final sequence of the synthetic DNA fragment is shown in the Supplementary Materials. For the construction, the *Xba*I/*Hin*dIII hydrolyzed *QHS1* fragment was inserted into likewise hydrolyzed pRhon5Hi-2 as well as a variant, providing the additional isoprenoid biosynthetic gene *ispA*. Thereby, the expression vectors pRhon5Hi-2-QHS1 and pRhon5Hi-2-QHS1-ispA were constructed, carrying the terpene synthase gene immediately downstream of the P*_nif_* promoter of the vector. Correct nucleotide sequences of all constructs were confirmed by Sanger sequencing (Eurofins Genomics, Ebersberg, Germany). The *QHS1* expression vectors are summarized in Table S1 (Supplementary Materials).

### 2.3. Cultivation of R. capsulatus for Heterologous Terpene Production

For the expression of the heterologous terpene biosynthetic genes, respective pRhon5Hi-2-based plasmids were transferred to cells of different *R. capsulatus* strains via conjugational transfer employing *E. coli* S17-1 as donor [84]. Thereafter, transconjugants were selected and further cultivated on PY agar containing kanamycin (25 µg mL^−1^) and rifampicin (25 µg mL^−1^). Subsequently, *Rhodobacter* cells were cultivated in airtight 4.5 mL screw neck vials (Macherey-Nagel, Düren, Germany) or airtight 15 mL hungate tubes [87] in liquid RCV medium containing kanamycin (25 µg mL^−1^) and rifampicin (25 µg mL^−1^). Precultures were cultivated in 15 mL RCV medium containing 0.1% (NH_4_)_2_SO_4_ inoculated with cells from a freshly grown PY agar plate and incubated for 48 h at 30 °C and with bulb light illumination. Expression cultures were inoculated from precultures to an optical density at 660 nm of 0.05 in 4.5 mL or 15 mL RCV medium containing 0.1% serine as an exclusive nitrogen source. Subsequently, cells were incubated at 30 °C under permanent illumination with bulb light (3.6 mW cm^−2^ at 850 nm) or IR light (5.6 mW cm^−2^ at 850 nm) for 3–5 days. For microaerobic expression cultures, cells were cultivated in 20–60 mL RCV medium containing 0.1% serine in 100 mL flasks at 30 °C and 130 rpm in the dark. The absence of ammonium and the cultivation under oxygen-limited conditions led to the induction of the P*_nif_*-dependent target gene expression. For the extraction of the produced sesquiterpenes, the cultures were overlaid with 150 µL or 500 µL *n*-dodecane, respectively, during inoculation [88]. 

### 2.4. Extraction, GC Analysis and Quantification of Sesquiterpenes

Basically, analysis of produced sesquiterpenes was conducted as described in Troost et al., 2019 [39]. In the following, the procedure is briefly described. To facilitate terpene extraction into the organic phase (*n*-dodecane) after cultivation, screw neck vials or hungate tubes were incubated in a horizontal position under permanent shaking (130 rpm, 30 °C, 24 h, in the dark) using a Multitron Standard incubation shaker (Infors HT). The *n*-dodecane samples were analyzed by gas chromatography (GC) using the Agilent *6890N* gas chromatograph equipped with a (5%-phenyl)-methylpolysiloxane *HP-5* column (length, 30 m; inside diameter, 0.32 mm; film thickness, 0.25 μm; Agilent Technologies) and a flame ionization detector (FID). The temperatures of the injector and FID were set to 240 and 300 °C, respectively. The GC was loaded with a 4-μL sample of each *n*-dodecane layer using a split ratio of 100:1 with helium as carrier gas. The following column temperatures were used during analysis: (i) 100 °C for 5 min, (ii) increased of temperature with a heating rate of 10 °C per min up to 180 °C, (iii) increased of temperature with a heating rate of 20 °C per min up to 300 °C. The signal of β-caryophyllene produced in *R. capsulatus* was verified by comparison of its retention times to a corresponding reference (β-caryophyllene from Sigma Aldrich, product number: 22075, retention time: 10.13 min). In order to determine the final product titers, the transfer efficiency from producing cells into the *n*-dodecane phase was determined as described in Supplementary Method section “Analysis of *n*-dodecane-mediated β-caryophyllene extraction from phototrophically grown *R. capsulatus*”. In brief, accumulated terpenes were extracted from cell lysates using *n*-dodecane. Subsequently, products were quantified using calibration curves of the reference compound, taking into account the specific transfer efficiencies of β-caryophyllene.

### 2.5. Effect of β-Caryophyllene and β-Caryophyllene Oxide on Plant Pathogenic Fungi

Isolates of the plant pathogenic fungi *P. lingam*, *S. sclerotiorum* and *A. brassicicola* were obtained from the Leibniz-Institut DSMZ (Deutsche Sammlung von Mikroorganismen und Zellkulturen GmbH, Braunschweig, Germany), while isolates of *F. oxysporum*, *R. solani* were obtained from the INRES, Plant Diseases and Plant Protection, University of Bonn. All isolates were sub-cultured on potato dextrose agar (PDA) at 24 °C and were used in this study to evaluate the bioactivity of the compounds on hyphal growth.

To test the bioactivities of β-caryophyllene and β-caryophyllene oxide, compounds were dissolved in a mixture of DMSO and Tween 20 (ratio of 1:2) to prepare differently concentrated stock solutions. These were mixed with PDA to gain the final concentrations 62.5, 125, and 250 µg mL^−1^ and to prepare PDA agar plates with terpenoids. The final DMSO and Tween 20 concentrations were always 1% (*v*/*v*) and 0.5% (*v*/*v*), respectively. Fungal discs with a diameter of 0.5 cm were cut from the culture media of freshly grown agar plates without terpenoids and placed upside down in the middle of PDA plates containing the chemicals. PDA plates with 0.5% (*v*/*v*) DMSO and 1% (*v*/*v*) Tween 20 alone were used as control. All plates were incubated for 7 days at 24 °C. Subsequently, the diameter of the fungal colony was measured, and the percentage of growth inhibition compared to the solvent control was calculated. Differences between the treatments were statistically analyzed using SigmaPlot software by one-way analysis of variance (ANOVA) and multiple comparisons for significance were performed at (*p* < 0.05) using the Holm-Sidak method.

### 2.6. Determination of the Minimum Inhibitory Concentration (MIC) of β-Caryophyllene and β-Caryophyllene Oxide in Liquid Cultures of Bacteria

The minimum inhibitory concentration of β-caryophyllene and β-caryophyllene was determined according to reference [89]. For the precultures, 10 mL Müller Hinton (MH) medium (Merck, Germany) was first inoculated in 100 mL flasks with four single bacterial colonies. For *R. capsulatus*, RCV was used. The liquid cultures were incubated for 18 h at 130 rpm and 37 °C (*R. capsulatus* at 30 °C). For the main cultures, MH or RCV medium was supplemented with differently concentrated stock solutions of β-caryophyllene and β-caryophyllene oxide in a mixture of DMSO and Tween 20 (ratio 1:2) to gain final concentrations of 62.5, 125, and 250 µg mL^−1^. All bacterial cultures were adjusted to a cell density corresponding to an optical density at 625 nm of 0.1 and then diluted 50-fold with medium for *B. subtilis*, *P. putida*, *P. fluorescens*, *R. rhizogenes* and *P. polymyxa* and 2-fold for *R. capsulatus*. For the inoculation of 96-well microtiter plates (Greiner Bio-One GmbH, Frickenhausen, Germany), 50 μL MH medium with the corresponding concentration of the substance to be tested and the solvent controls were mixed with 50 μL of previously diluted bacterial culture, resulting in an end optical density at 625 nm of 0.001 and 0.025, respectively. The solvent control contained 1% (*v*/*v*) Tween 20 and 0.5% (*v*/*v*) DMSO. After inoculation, the microtiter plates (MTPs) were first shaken in a SpectraMax i3x (Molecular Devices, San Jose, CA, USA) plate photometer for 20 s to mix the solution and then incubated for 20 h at 37 °C. *R. capsulatus* was incubated at 30 °C and 300 rpm. For subsequent determination of the MICs, the optical density of cell cultures was determined at 625 nm in a plate photometer. The MIC was defined based on the European Committee on Antimicrobial Susceptibility Testing (EUCAST) guidelines as the compound concentration at which an optical density at 625 nm minus the background absorbance equals 0 [90].

## 3. Results

In the past, terpenoids were exclusively obtained from natural plant sources, e.g., by extracting them from essential oils, requiring a complex and time-consuming downstream processing. The heterologous production of sesquiterpenes in a suitable microbial host, however, bears many benefits. For example, it offers the possibility to solely produce a desired compound so that it can be rather easily purified without the need of removing closely related constituents [21,34]. Thus, we here aimed to reconstitute the plant sesquiterpene pathway of β-caryophyllene in *R. capsulatus* and optimize the production under phototrophic growth conditions. Since many sesquiterpenoids exhibit promising antimicrobial activities, the antifungal efficacy of β-caryophyllene and its oxidized form against phytopathogenic fungi were evaluated.

### 3.1. Establishment of β-Caryophyllene Production in R. capsulatus via Overexpression of Isoprenoid Precursor Genes

**Figure 1:**
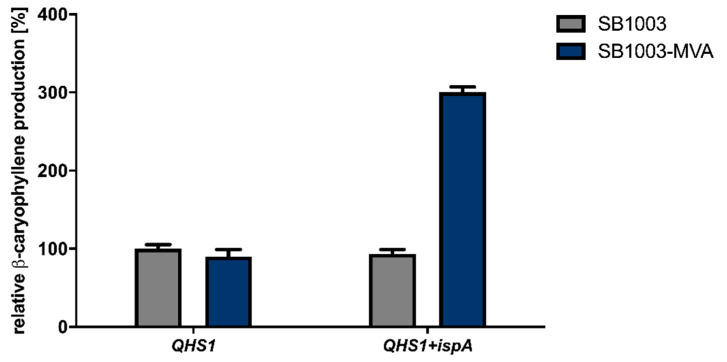
Heterologous β-caryophyllene production in the *R. capsulatus* strains SB1003 and SB1003-MVA. The β-caryophyllene synthase gene *QHS1* from *A. annua* was expressed in *R. capsulatus* SB1003 wild type (grey bars) and SB1003-MVA (blue bars), which additionally carries the MVA gene cluster from *P. zeaxanthinifaciens* to enable a second isoprenoid biosynthesis route. Moreover, the *ispA* gene encoding the *R. capsulatus* FPP synthase was co-expressed on the same plasmid to further enhance sesquiterpene production titers. Product formation was determined in cell cultures after three days of photoheterotrophic cultivation (gas-tight hungate tubes, 30 °C) under ammonium depletion and constant illumination with bulb light (3.6 mW cm^−2^ at 850 nm). The produced β-caryophyllene was sampled in overlaid *n*-dodecane phases for GC-FID analysis. The increase of β-caryophyllene production in engineered *R. capsulatus* strains is shown as relative values. To this end, the *R. capsulatus* SB1003 carrying the plasmid-encoded *QHS1* gene was used as a reference strain. Values are means of three independent biological replicates (*n* = 3) and error bars indicate the respective standard deviations.

Recently, we described the heterologous synthesis of the plant sesquiterpenoids valencene and patchoulol in the phototrophic bacterium *R. capsulatus* and its modular improvement by engineering the biosynthesis of the central precursor FPP [39]. To evaluate if *R. capsulatus* and the modular engineering principle can analogously be applied for the synthesis of the plant-derived sesquiterpene β-caryophyllene, we expressed the gene encoding β-caryophyllene synthase QHS1 from *A. annua* in the bacterial host. To this end, the expression vectors pRhon5Hi-2-QHS1 and pRhon5Hi-2-QHS1-ispA, carrying an additional copy of the intrinsic FPP synthase gene *ispA*, were transferred to the *R. capsulatus* wild type strain SB1003 and strain SB1003-MVA. The latter strain additionally contains the chromosomally integrated MVA pathway genes derived from *Paracoccus zeaxanthinifaciens* and thus offers a second isoprenoid biosynthesis pathway. To compare the β-caryophyllene production in all *Rhodobacter* strains grown under phototrophic conditions, cells were incubated in the absence of molecular oxygen and ammonium under constant bulb light illumination. Terpene accumulation was determined in the late stationary growth phase by analyzing *n*-dodecane samples via GC-FID measurements. The increase of β-caryophyllene production in tested *R. capsulatus* strains is shown in Figure 1 as relative values using *R. capsulatus* SB1003 solely carrying the plasmid-encoded *QHS1* gene as reference strain.

As shown in Figure 1, the expression of the β-caryophyllene synthase gene *QHS1* in *R. capsulatus* strain SB1003 led to a measurable production of β-caryophyllene. Remarkably, the co-expression of *QHS1* and *ispA* in the *R. capsulatus* strain SB1003 as well as *QHS1* expression in the engineered SB1003-MVA strain did not result in increased β-caryophyllene synthesis. However, concerted expression of *QHS1* and *ispA* in *R. capsulatus* SB1003-MVA led to a considerable increase of sesquiterpenoid production of about 300% in comparison to the reference strain.

### 3.2. Optimization of β-Caryophyllene Production in R. capsulatus via Modification of Cultivation Conditions

**Figure 2:**
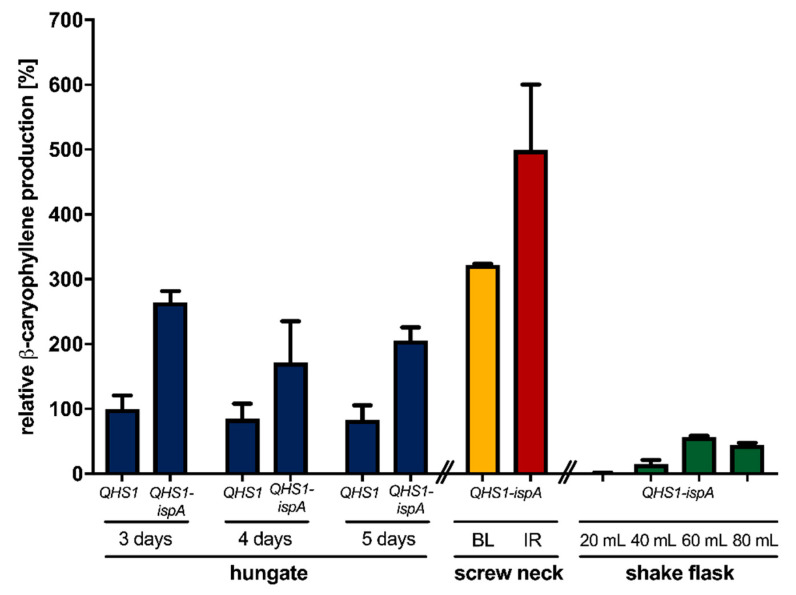
Heterologous β-caryophyllene production in the *R. capsulatus* strain SB1003-MVA with dependence on the cultivation time and illumination conditions. The β-caryophyllene accumulation was determined in *R. capsulatus QHS1* expression strains SB1003-MVA (pRhon5Hi-2-QHS1) and SB1003-MVA (pRhon5Hi-2-QHS1-ispA). First, product formation was determined in cell cultures after three, four, and five days of photoheterotrophic cultivation in 15 mL hungate tubes using standard illumination conditions (bulb lights, 3.6 mW cm^−2^ at 850 nm) and RCV medium supplemented with 0.1% serine. Blue bars represent the results of this experiment. Second, illumination conditions were changed by cultivating *R. capsulatus* strain SB1003-MVA (pRhon5Hi-2-QHS1-ispA) for three days under photoheterotrophic conditions using either constant illumination with bulb lights (BL; 3.6 mW cm^−2^ at 850 nm, yellow bar) or IR-emitting diodes (IR; 5.6 mW cm^−2^ at 850 nm, red bar). Here, 4.5-mL screw neck vials were used to improve light penetration due to a more favorable surface-area-to-volume ratio of this cultivation vessel. For non-phototrophic cultivation, the same strain was grown in 100-mL, unbaffled shake flasks containing different volumes of serine-supplemented RCV medium (shake flask, green bars). In all cultures, the produced β-caryophyllene was sampled in overlaid *n*-dodecane phases for GC-FID analysis. The increase of β-caryophyllene production is shown as relative values using *R. capsulatus* SB1003-MVA carrying the QHS1 expression plasmid pRhon5Hi-2-QHS1 as a reference strain. Values are the means of three independent biological replicates (*n* = 3) and the error bars indicate the respective standard deviations.

In the above-described experiments, we could demonstrate that modular engineering of the isoprenoid biosynthesis can also be applied to improve β-caryophyllene production in *R. capsulatus*. Next, we analyzed whether the modification of cultivation conditions including a prolonged cultivation time or the change of illumination parameters can further improve the product yield in the better-performing strain SB1003-MVA. First, β-caryophyllene accumulation was comparatively analyzed over five days in photoheterotrophically-grown cultures of *R. capsulatus* strain SB1003-MVA carrying pRhon5Hi-2-QHS1 or pRhon5Hi-2-QHS1-ispA. Product formation was determined by analyzing the overlaid *n*-dodecane samples via GC-FID measurements (Figure 2, blue bars).

The highest product levels could be detected after three days of cultivation, where cells have typically reached the beginning of the stationary growth phase. The elongation of the cultivation time did not show increased product accumulation so that all further production experiments were carried out for three days. Under standard phototrophic conditions, conventional light bulbs are used for the illumination of *R. capsulatus* cells [39,49]. This conventional light source offers a broad emission spectrum with a relatively high proportion in the infrared (IR) light range (>750 nm; 3.6 mW cm^−2^ at 850 nm) suitable for excitation of bacteriochlorophyll *a* (BChl *a*) exhibiting excitation maxima at 800 and 860 nm (Figure S6, Supplementary Materials, Reference [91]). To improve the illumination conditions for sesquiterpene production under phototrophic conditions, we subsequently analyzed if the use of (i) alternative cultivation vessels offering a better light penetration of cell cultures by a more favorable surface-area-to-volume ratio (Table S2, Supplementary Materials) or (ii) a customized IR-LED array (5.6 mW cm^−2^ at 850 nm) suitable for specific excitation of the photopigment BChl *a* with high light intensities can help to increase product formation.

To investigate the influence of illumination conditions on the heterologous production of β-caryophyllene, the strain SB1003-MVA carrying the expression vector pRhon5Hi-2-QHS1-ispA was cultivated over three days under photoheterotrophic conditions and constant illumination with bulb light or IR light in an ammonium-depleted medium in screw neck vials. As shown in Figure 2, the change of cultivation vessel geometry resulted only in a slight increase of β-caryophyllene production of *R. capsulatus* SB1003-MVA (pRhon5Hi-2-QHS1-ispA), whereas high irradiation with IR light led to a 1.9-fold increase of the final product accumulation. These results indicate that the applied illumination conditions should be taken into account to reach high product yields when *R. capsulatus* is used as an alternative terpene production host. This assumption is further supported by the observation that product levels were much lower in *R. capsulatus* SB1003-MVA (pRhon5Hi-2-QHS1-ispA) cultures that have been grown under non-phototrophic, i.e., microaerobic conditions (Figure 2, green bars). For non-phototrophic cultivation, *R. capsulatus* SB1003-MVA (pRhon5Hi-2-QHS1-ispA) was grown in 100 mL, unbaffled shake flasks containing different volumes of medium in the dark to implement different aeration conditions (green bars). Those conditions could lead to the formation of β-caryophyllene oxide, the oxygenated derivative of β-caryophyllene. As previously described, a filling volume of 60 mL is most suitable for the induction of intrinsic terpene formation and P*_nif_*-mediated target gene expression in *R. capsulatus* [39], which is corroborated by the observed β-caryophyllene production levels. Nevertheless, only a quarter of the product yield could be achieved under microaerobic, non-phototrophic growth conditions when compared to the corresponding values of phototrophically grown cells (*R. capsulatus* SB1003-MVA, pRhon5Hi-2-QHS1-ispA, 3 days, bulb light), and only traces of the oxygenated derivative were detectable (data not shown). However, to fully understand the effects of varying cultivation conditions on β-caryophyllene production, further experiments have to be performed in future studies.

To accurately determine the final product titers, we analyzed (i) the individual transfer efficiency of β-caryophyllene from intact cells into the *n*-dodecane phase, (ii) the effect of the ICM, which is formed by *R. capsulatus* cells under phototrophic conditions, on sesquiterpenoid extraction, (iii) the differences in terpene transfer efficiencies when comparing single and repeated *n*-dodecane extraction, and finally (iv) the effect of the presence and absence of organic solvent on the final product titers (Supplementary Method section “Analysis of *n*-dodecane-mediated β-caryophyllene extraction from phototrophically grown *R. capsulatus*”). Finally, we were able to determine a product titer of 90 ± 19 mg L^−1^ β-caryophyllene for *R. capsulatus* SB1003-MVA with pRhon5Hi-2-QHS1-ispA after 3 days of cultivation in hungate tubes under bulb light. This titer could be further increased by using IR light and screw neck vials for cultivation, reaching a final product titer of 139 ± 31 mg L^−1^. Based on these values and the reached cell densities, the respective productivities were further calculated (Table S3, Supplementary Materials).

In summary, we showed that *R. capsulatus* can efficiently synthesize the sesquiterpene β-caryophyllene. Furthermore, the modular adaptation of precursor gene expression under phototrophic growth conditions as well as the adjustment of cultivation conditions resulted in an increased sesquiterpenoid formation.

### 3.3. Evaluation of Bioactivities of β-Caryophyllene and β-Caryophyllene Oxide against Different Phytopathogenic Organisms

The agricultural industry is affected by a dwindling number of effective antimicrobial substances. On the other hand, farmers have to control plant pathogenic organisms without damaging non-target organisms. As β-caryophyllene and β-caryophyllene oxide offer a variety of beneficial bioactivities [70,79,92,93,94,95], we evaluated the potential use of those two sesquiterpenes as a nature-derived fungicide. To this end, we analyzed the activity against different phytopathogenic fungi, as well as various plant growth-promoting bacteria (PGPB).

#### 3.3.1. Bioactivities of β-Caryophyllene and β-Caryophyllene Oxide against Phytopathogenic Fungi

**Figure 3:**
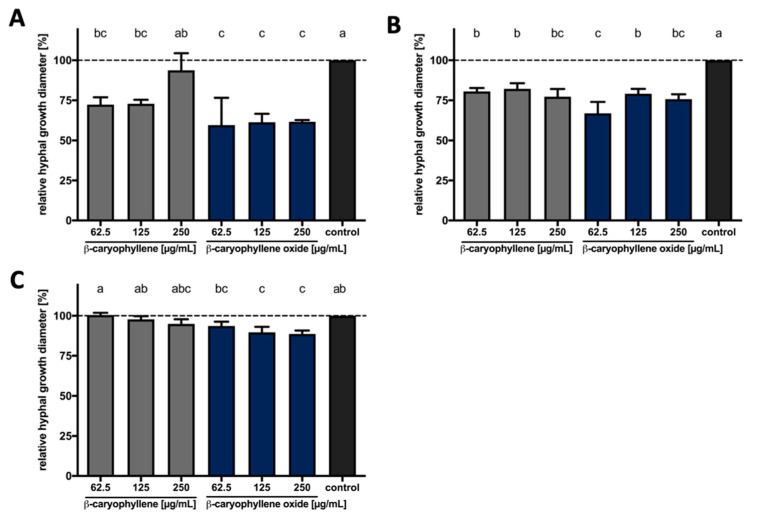
The effect of β-caryophyllene and β-caryophyllene oxide on the hyphal growth of plant pathogenic fungi. The effect of β-caryophyllene and β-caryophyllene oxide against *S. sclerotiorum* (**A**), *F. oxysporum* (**B**) and *A. brassicicola* (**C**). Final concentrations of 62.5 µg mL^−1^, 125 µg mL^−1^, and 250 µg mL^−1^ of β-caryophyllene (grey bars) and β-caryophyllene oxide (blue bars) in PDA growth medium were used. Medium mixed with the solvents DMSO and Tween 20 (final concentrations, 0.5% and 1% *v*/*v*, respectively) was used as the control (black bars). An equally sized disk with fungal mycelium was placed in the center of each plate and incubated for seven days at 24 °C. Subsequently, the diameter of each fungal colony was measured, and the relative growth compared to the solvent control was calculated. Each bar represents the mean  ±  standard deviation of three independent biological measurements with three technical replicates each (*n*  =  9). Different letters on the top of the bars indicate significant differences between the treatments based on ANOVA and Holm-Sidak post-hoc method (*p*  <  0.05), while the same letters represent no significant differences.

We investigated the bioactivity of β-caryophyllene and β-caryophyllene oxide, which can be formed spontaneously by uncatalyzed processes [96,97], against the plant pathogenic fungi *S. sclerotiorum*, *F. oxysporum*, *A. brassicicola*, *P. lingam*, and *R. solani*. This analysis would additionally reveal whether the compound’s oxygenation influences potential antifungal properties. Therefore, PDA agar plates were supplemented with increasing concentrations of both compounds, fungal discs were transferred onto these plates and fungal growth was determined. Evaluation revealed that the degree of growth inhibition due to direct terpene exposure varied depending on the compound and the fungus (Figure 3).

Both compounds inhibited the hyphal growth of *S. sclerotiorum* when compared to the solvent control. The inhibition reached up to 30% when the fungus was exposed to β-caryophyllene, while it was up to 40% when the fungus was cultivated on medium containing β-caryophyllene oxide. The effect against *F. oxysporum* was less pronounced. Around 20% inhibition was observed when the fungus was cultivated on the β-caryophyllene-supplemented medium, while it was around 30% in the case of β-caryophyllene oxide. Finally, the presence of β-caryophyllene in the growth medium slightly inhibited the growth of *A. brassicicola* while inhibition was higher and reached a maximum of 10% when β-caryophyllene oxide was used. No significant effect of both compounds was observed against *P. lingam* and *R. solani* (Figure S7, Supplementary Materials). Our results thus reveal that β-caryophyllene and its oxidized form possess antifungal activity against certain phytopathogenic fungi and that β-caryophyllene oxide tends to be more effective in inhibiting fungal growth.

#### 3.3.2. Antimicrobial Activities against Plant Growth-Promoting Bacteria

**Figure 4:**
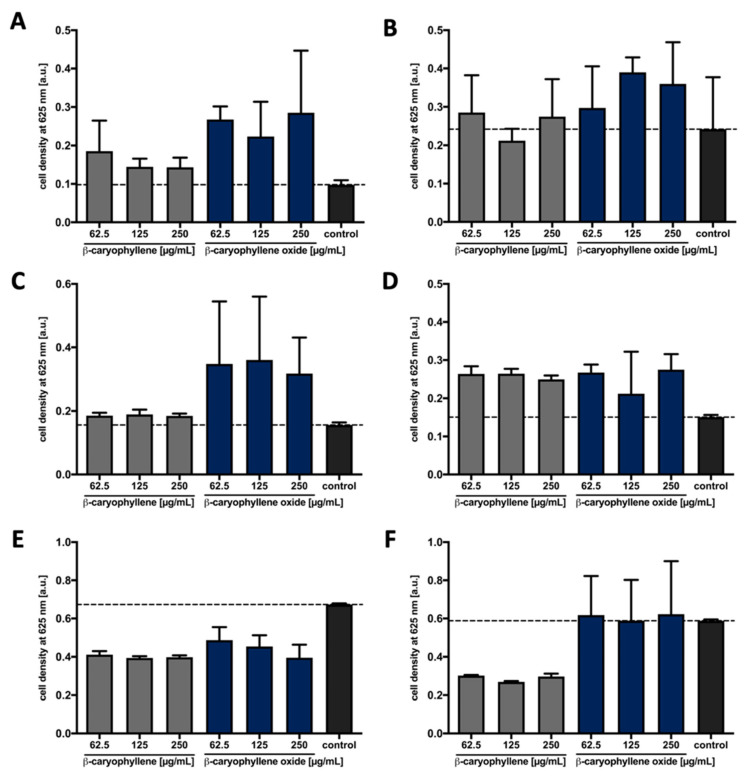
The influence of β-caryophyllene and β-caryophyllene oxide on the growth of plant growth-promoting bacteria. Final concentrations of 62.5 µg mL^−1^, 125 µg mL^−1^ and 250 µg mL^−1^ of β-caryophyllene (grey bars) and β-caryophyllene oxide (blue bars) were added to cultures of *R. rhizogenes* (**A**), *R. capsulatus* (**B**), *B. subtilis* (**C**), *P. polymyxa* (**D**), *P. fluorescens* (**E**) and *P. putida* (**F**) in 100 µL MH medium (*R. capsulatus* in RCV medium) in MTPs. The final solvent concentration was 1% (*v*/*v*) Tween 20 and 0.5% (*v*/*v*) DMSO. To determine the influence of the terpenes on the growth of the bacteria, the cells were incubated stationary for 20 h at 37 °C (*R. capsulatus* at 30 °C) and the cell density was measured at 625 nm using a plate photometer. The solvent control (control, black bars) was MH or RCV medium containing 1% (*v*/*v*) Tween 20 and 0.5% (*v*/*v*) DMSO. Values are means of three independent biological replicates (*n* = 3) and error bars indicate the respective standard deviations.

As the previous investigations showed antifungal properties against several phytopathogenic fungi, the use of the sesquiterpenoids as natural compound-based plant protection products could be considered. To investigate potential toxic off-target effects on bacteria that promote plant growth, we next examined whether the addition of β-caryophyllene/oxide affects the growth of bacteria at concentrations used in the hyphal growth assay. For this purpose, the growth of representatives of the plant growth-promoting bacteria (PGPB) group [98,99,100], including the two diazotrophic bacteria *Rhizobium rhizogenes* and *Rhodobacter capsulatus* [101,102], the two bacilli *Bacillus subtilis* [103,104] and *Paenibacillus polymyxa* [105], as well as the pseudomonads *Pseudomonas fluorescens* [104] and *Pseudomonas putida* [106] was analyzed in presence of β-caryophyllene and β-caryophyllene oxide. Both compounds were added to diluted bacterial cultures in increasing concentrations. After overnight incubation, the MICs were determined according to the respective optical density of the cell cultures (Figure 4).

The bacteria *R. rhizogenes*, *R. capsulatus*, *B. subtilis*, and *P. polymyxa* did not show reduced cell growth in comparison to the solvent control upon the addition of the two terpenes β-caryophyllene and β-caryophyllene oxide (Figure 4A–D). These bacteria showed an increase in growth, which could be explained by the metabolization of the terpenes. For *P. putida*, no effect of β-caryophyllene oxide was detected compared to the solvent control (Figure 4F). β-caryophyllene showed an influence on *P. putida*, which was concentration independent since all tested concentrations led to comparable cell growth. The cell densities were about 40% lower compared to the solvent control. This effect was also observed for *P. fluorescens*, where the two terpenes reduced growth by up to 40% (Figure 4E).

In summary, for β-caryophyllene and β-caryophyllene oxide, no MIC could be determined for any of the tested PGPB, but a reduction of cell growth could be observed for both Pseudomonads. As a diverse group of different representative soil bacteria was tested, the results nevertheless indicate that β-caryophyllene and β-caryophyllene oxide do not exhibit strong broad-spectrum antibacterial activities at concentrations which considerably inhibit the hyphal growth of *S. sclerotiorum* and *F. oxysporum* (63 µg mL^−1^).

## 4. Discussion

The management of plant pathogens in the process of crop production is crucial, no matter whether organic, integrated, or conventional farming practices are applied. For many decades, synthetic pesticides were considered the fastest and most effective pest and pathogen control method. Recently, due to the rise of public health concerns about pesticide toxicity and harm to the environment, many of these effective chemicals were banned, thereby markedly limiting the options for plant protection. Therefore, it is important to find environmentally safe and sustainable natural products to control pathogens and thus ensure yield and food quality. In this context, plant metabolites are a rich source of bioactive compounds explorable for the use of preventing and controlling plant pathogenic microbes. In the last decade, several studies investigated terpenoids as potential antiphytopathogenic compounds [67,71,76,77,107,108]. β-caryophyllene is a natural bicyclic sesquiterpene that is a constituent of many essential oils. Many studies showed that these essential oils, which are containing β-caryophyllene as one of the main ingredients, are active against plant pathogens [109,110,111]. For example, methanol extracts from *Artemisia annua* leaves, one of the common β-caryophyllene producers, strongly inhibit the growth of the plant pathogenic fungi *F. oxysporum* and *Fusarium solani* [79]. In another study, the essential oil from *Murraya paniculata* leaves showed inhibitory activities on the mycelial growth of *S. sclerotiorum*, a fungus that poses a high risk to several crops. The gas chromatography analysis of the essential oil composition introduced β-caryophyllene as one of the main constituents (23.8%) [109]. Furthermore, essential oils from *Piper aduncum*, which also has β-caryophyllene as one of its main constituents (7.2%), inhibited the mycelial growth of the fungus *S. sclerotiorum* [110]. As a second alternative or complementary means for plant protection, there is also a multitude of important and useful microorganisms that support plant growth, which are called plant growth-promoting bacteria and plant growth-promoting fungi (PGPF). To offset the negative effects of chemical substances or make their use superfluous, more and more PGPB are now being used in agriculture [112]. Microorganisms can fulfill different functions in this process. *Bacillus subtilis*, for example, accumulates at the root system during the germination of various plants and prevents competing harmful fungi from spreading [103]. Diazotrophic organisms can supply plants with biologically available nitrogen by fixing atmospheric dinitrogen, thus making it available to the plants [113]. When fighting phytopathogens, it is important to consider and ideally avoid negative off-target effects on the above-mentioned beneficial microorganisms. Corresponding tests are therefore now frequently included in the first evaluation of antimicrobial activities.

So far, no studies were investigating the effect of pure β-caryophyllene and β-caryophyllene oxide against a selection of phytopathogenic fungi aiming to determine and compare the potential antifungal properties of the two compounds and species-specific differences in sensitivity. In our current study, we show the potential of sustainable production of β-caryophyllene in the heterologous host *Rhodobacter capsulatus* and the species-specific promoting or inhibitory effects for selected plant growth-promoting bacteria for both of the tested sesquiterpenoids at appropriate compound concentrations. Furthermore, we tested the bioactivities of both β-caryophyllene and β-caryophyllene oxide against several plant pathogenic fungi and showed that both substances were active against certain fungi. Interestingly, the oxidized form tended to be even more effective, and additionally has a more beneficial activity profile concerning the PGPB. These results are supported by previous reports which are introducing β-caryophyllene as a bioactive compound in its purified form [79] and as a component of several essential oils [109,110]. The purified β-caryophyllene showed a MIC of 130 µg mL^−1^ for *F. oxysporum* [79], which is below the maximal concentration tested in this study. However, our plate-based approach is not completely comparable with the method used for MIC determination in liquid medium. According to our results, the inhibitory effect of the hyphal growth was different depending on the tested fungus. Such a difference is dependent on the fungal species and frequently described by previous studies showing that the novel fungicide 3-[5-(4-chlorophenyl)-2,3-dimethyl-3-isoxazolidinyl] pyridine (SYP-Z048) affected several pathogenic fungi in different ways [114]. Overall, our current results demonstrate that both β-caryophyllene and β-caryophyllene oxide exhibit bioactivity against plant pathogenic fungi and therefore could be suitable as potential fungicides in agriculture as, in contrast to many broad-spectrum pesticides, they do not harm many species of plant growth-promoting bacteria. However, despite the sesquiterpenes being natural compounds, which are often associated with non-harmful ecotoxicological profiles, effects against *Pseudomonas* species were corroborated and will need to be taken into account. So far, there is only limited information about the individual activities of terpenes against plant pathogens and the underlying molecular mechanisms. To be able to explain the differences we observed in the activity of the two terpenes against the different organisms and to get more data on the activity spectrum, our investigations need to be extended by including more target and non-target organisms. In addition, the respective modes of action on the molecular level have to be determined. Besides additional plant pathogens, this not only includes analyzing further plant growth-promoting bacteria, but it must be tested if plant growth-promoting fungi react sensitively to the terpenes, as indicated by a previous promising study [80]. In particular, fungi of the genus *Trichoderma*, which are said to have many advantageous properties for plants, could be investigated more closely [115,116].

To be able to provide appropriate quantities of an active antifungal substance, the heterologous production of promising sesquiterpenes in a suitable microbial host bears various benefits, such as the possibility to solely produce the desired compound without complex downstream processing and in high amounts. Therefore, we established the biosynthesis of the plant sesquiterpene β-caryophyllene in the heterologous production host *R. capsulatus* under phototrophic and non-phototrophic conditions. For this purpose, the intrinsic isoprenoid biosynthesis pathway was optimized in terms of its precursor supply. In particular, the P*_nif_*-based co-expression of *ispA* and the genetically integrated MVA pathway resulted in a substantial increase in sesquiterpenoid production of around 300%. These results are in agreement with previous studies, where engineering of isoprenoid precursor supply was a valuable tool to increase the terpenoid production in *Rhodobacter* [38,40,48] and other bacterial hosts [23,117,118,119,120,121,122,123,124,125]. Also, we were able to increase the terpene production level further by changing the cultivation conditions from bulb light in a 14 mL hungate tube to IR light in a 4.5 mL screw neck vial, achieving a final β-caryophyllene titer of 139.29 ± 31.35 mg L^−1^ and a specific productivity of 1.30 ± 0.32 mg g^−1^ dry cells h^−1^. In recent studies, production titers around 220 mg L^−1^ [126] and specific productivities of 1.15 mg g^−1^ dry cells h^−1^ [127] were achieved in *E. coli*. Thus, we attained yields comparable to the current literature and successfully established *R. capsulatus* as a heterologous host for the production of β-caryophyllene. Furthermore, the β-caryophyllene yields achieved in *R. capsulatus* could be sufficient to use this host as a microbial system for in situ agent delivery. In the future, sesquiterpenoid producing *R. capsulatus* might thus be applicable as cell extracts with biocontrol activities for plant protection or as engineered antiphytopathogenic PGPB that can be added as live cultures to soils contained in vertical farming.

## Data Availability

The datasets generated and/or analyzed during the current study are available from the corresponding authors on reasonable request.

## Supplementary Materials

The following are available online at https://www.mdpi.com/2076-2607/9/1/168/s1. Figure S1: Transfer efficiency of the β-caryophyllene reference compound from cultivation medium into the *n*-dodecane phase in the presence of intact *R. capsulatus* cells in hungate and screw neck vials, Figure S2: Transfer efficiency of the β-caryophyllene reference compound from cultivation medium into the *n*-dodecane phase in the presence of intact and disrupted *R. capsulatus* cells, Figure S3: Extraction efficiency of the β-caryophyllene reference compound from cultivation medium in the presence of disrupted and intact *R. capsulatus* cells by repeatedly using *n*-dodecane as organic solvent over four days, Figure S4: Comparison of relative β-caryophyllene formation in *R. capsulatus* production strains cultivated with and without an *n*-dodecane layer, Figure S5: Quantification of extracted β-caryophyllene via a calibration curve of β-caryophyllene reference signals in GC-FID analyses, Figure S6: Emission range of different light sources and the absorption spectrum of phototrophically cultivated *R. capsulatus* cells [128], Figure S7: Effect of β-caryophyllene and β-caryophyllene oxide on the hyphal growth of plant pathogenic fungi, Table S1: Bacterial strains and plasmids used in this study, Table S2: Cultivation vessel specifications, Table S3: Productivities of β-caryophyllene in *R. capsulatus* SB1003 cultures.
