# Supplementary material for: Heterologous Production of β-Caryophyllene and Evaluation of Its Activity against Plant Pathogenic Fungi
Source: Microorganisms. 2021 Jan 14;9(1):168. doi: 10.3390/microorganisms9010168 (PMC7828715; doi:10.3390/microorganisms9010168)
Supplement: Supplementary file 1 [file microorganisms-09-00168-s001.pdf]

Supplementary Materials

Heterologous Production of β-Caryophyllene and Evaluation of its Activity against Plant Pathogenic Fungi

Table of contents

1 Supplementary methods 2

Bacterial strains and plasmids 2

Analysis of *n*-dodecane-mediated β-caryophyllene extraction from phototrophically grown *R. capsulatus*. 3

2 Supplementary data 7

Emission range of used light sources and *in vivo* absorption spectrum of phototrophically grown *R. capsulatus* culture 7

Specifications of cultivation vessels for anaerobic growth of *R. capsulatus* cultures. 7

Productivities of β-caryophyllene in *R. capsulatus* SB1003 cultures. 8

Codon optimized DNA sequence of β-caryophyllene synthase QHS1 from *A. annua* for expression in *R. capsulatus*. 9

Bioactivities of β-caryophyllene and β-caryophyllene oxide against phytopathogenic fungi 10

References 11

1. Supplementary methods

Bacterial strains and plasmids

All bacterial strains, plasmids and oligonucleotides used in this study are listed in Table S1.

Table S1. Bacterial strains and plasmids used in this study.

Strains, plasmids	Relevant features or description	References
	**Strains**	
***E. coli* DH5α**	*F⁻Φ80lacZΔM15 Δ(lacZYA-argF) U169 recA1 endA1 hsdR17 phoA supE44 thi-1 gyrA96 relA1 deoR*	[81]
***E. coli* S17-1**	Ec294::[RP4-2 (Tc^R::Mu)(Km^R::Tn7)] *recA, thi, pro, hsdR⁻ hsdM⁺* Tp^R Sm^R	[82]
***R. capsulatus* SB1003**	Wild-type, Rif^R	[83]
***R. capsulatus* SB1003-MVA**	SB1003-derivative, carrying the chromosomally intergrated MVA cluster from *Paracoccus zeaxanthinifaciens*, Rif^R, Gm^R	[39]
	**Plasmids**	
**pRhon5Hi-2**	pBBR1mcs (basic vector, rep mob Cm^R), pET22b (MCS, pelB), pBSL15 (aphII), P_{nif}	[39]
**pRhon5Hi-2-QHS1**	pRhon5Hi-2 with P_{nif} *QHS1*	This study
**pRhon5Hi-2-QHS1-ispA**	pRhon5Hi-2 with P_{nif} *QHS1-ispA*	This study

Analysis of *n*-dodecane-mediated β-caryophyllene extraction from phototrophically grown *R. capsulatus*.

The extraction of sesquiterpenoids from microbial cell cultures is mostly performed *via* a two-phase cultivation using an *n*-dodecane layer ($^1/_{30}$ of the culture volume) as solvent phase which is added prior to cultivation [88]. In analogy to our prior work [39], we performed the extraction and quantification of β-caryophyllene as described in the Materials and Methods section.

To quantify the final product titers, a calibration curve was generated using a pure β-caryophyllene reference from Sigma Aldrich. However, the correlation of signals from *n*-dodecane extracted samples with the reference signals does not take into account extraction efficiencies of β-caryophyllene when using *n*-dodecane as organic solvent. It can be assumed that, in dependence of its specific properties, it only diffuses to a certain extent into the *n*-dodecane layer. In addition, terpenes that are produced in the cytoplasm of *R. capsulatus* can additionally be retained by the intracytoplasmic membrane system thereby further affecting the transfer into the organic phase. Therefore, the transfer efficiency of β-caryophyllene from cultivation medium into *n*-dodecane in the presence of intact and disrupted *R. capsulatus* cells was first determined for both hungate and screw neck vials. For this purpose, the reference compound was first mixed with 14 mL or 4.5 mL of phototrophically grown *R. capsulatus* SB1003 cells (cultivation parameters: anaerobic growth, 30 °C, approx. up to OD_{660nm} = 2.5) in appropriate amounts (β-caryophyllene: 71.4 mg L^{-1} and 881.4 mg L^{-1}, respectively). For this purpose, 11.48 µL or 51.7 µL β-caryophyllene, which is an oil, was added as a 10-fold dilution in diethyl ether. After addition, the cultures were sealed and vortexed for 1 min. Subsequently, the reference substance was extracted using *n*-dodecane as described. The transfer efficiency was determined via GC analysis by comparing peak areas of the specific signals from appropriately diluted solutions to samples that had undergone extraction (**Figure S1**).

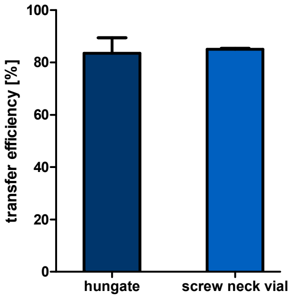

Figure S1. Transfer efficiency of the β-caryophyllene reference compound from cultivation medium into the *n*-dodecane phase in the presence of intact *R. capsulatus* cells in hungate (dark blue) and screw neck vials (light blue). For extraction, 71.4 mg L^{-1} or 881.4 mg L^{-1} β-caryophyllene were added to 14 mL or 4.5 mL cell culture (OD660nm = 2.5), respectively. For details, see text above. Values are means of triplicate measurements. Error bars indicate the respective standard deviations.

By using intact *R. capsulatus* cells, a transfer efficiency of around 85% could be determined for both the hungate and the screw neck vial. Thus, it could be shown that there are some methodological losses, which have to be considered for product quantification. To moreover analyze if putative interaction of intracellularly produced sesquiterpenoids with the *Rhodobacter* ICM can further decrease product transfer, the experiment was repeated using disrupted cells. However, as the transfer efficiencies were nearly equal for the hungate and the screw neck vial, all further experiments were only performed for the hungate cultivation. First, equally cultivated *R. capsulatus* wildtype cells (OD_{660nm} = 2.5) were disrupted using a ball mill (3 × 10 min, 30 Hz, Mixer Mill MM 400, Retsch GmbH, Germany) and subsequently mixed with the same amount of reference compound as described previously. Extraction

and quantification were performed as described for intact cell samples and signals were subsequently compared to those of the non-extracted reference compounds (**Figure S2**).

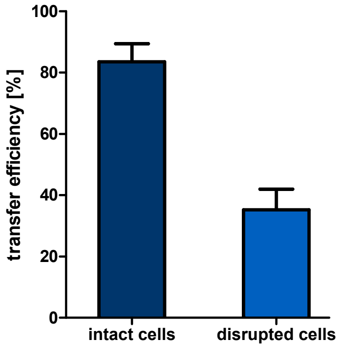

Figure S2. Transfer efficiency of the β-caryophyllene reference compound from cultivation medium into the *n*-dodecane phase in the presence of intact (dark blue) and disrupted (light blue) *R. capsulatus* cells. For extraction, 71.4 mg L^{-1} β-caryophyllene were added to 14 mL cell lysate (OD660nm = 2.5). For further details, see text above. Values are means of triplicate measurements. Error bars indicate the respective standard deviations.

It could be seen, that disrupted cells lead to a strong decrease of the extraction efficiency (35%) in comparison to the previous measurement using intact cells (85%), suggesting that this hydrophobic terpenoid (log*P* = 5.35; values were calculated using the ALOGPS2.1 online tool described by Tetko *et al.* 2005; doi:10.1007/s10822-005-8694-y) is strongly attached to the intracytoplasmic membrane system. Hence, for calculating the final production titers, the individual transfer efficiency for disrupted cell cultures (here termed c_t *or* 'transfer efficiency coefficient', factor: 1.6474) was taken into account.

Besides the above described negative effect of cellular components on the extraction efficiency, we further analyzed, if repeated *n*-dodecane-dependent sesquiterpenoid extraction should be considered for an optimal estimation of the overall production titers. Thus, an experiment with repeated sesquiterpenoid extractions from disrupted wildtype cultures that were mixed with the reference compound as described above was performed over four days (**Figure S3**). For quantitative analysis of β-caryophyllene, a calibration curve with the authentic reference compound ranging from 0.5 to 4 mg mL^{-1} *n*-dodecane, were used (slope: 382.16; see also depicted below in **Figure S5**).

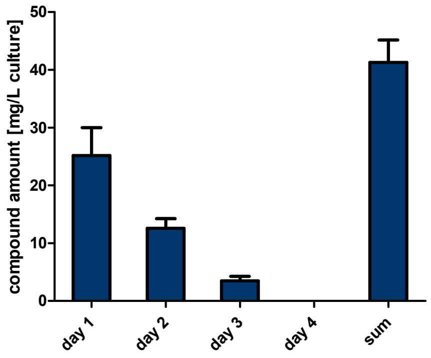

Figure S3. Extraction efficiency of the β-caryophyllene reference compound from cultivation medium in the presence of disrupted (dark blue) and intact (light blue) *R. capsulatus* cells by repeatedly using *n*-dodecane as organic solvent over four days. See text above for details. For repeated extraction, 71.4 mg L^{-1} β-caryophyllene were added to 14 mL cell lysate (OD660nm = 2.5). Subsequently, 500 μL *n*-dodecane was used for a 24 h extraction period over a total duration of four days. Single extraction procedures were repeated four times and the sesquiterpenoid concentration of each fraction was analyzed via GC. Values are means of triplicate measurements. Error bars indicate the respective standard deviations.

The overall extraction efficiency was increased by repeated extraction to 39% for disrupted cells in comparison to the amount determined after the first extraction. Therefore, it has also to be taken into account by which factor the quantification on day 1 underestimates product titers (here termed c_{ex} *or* 'coefficient for repeated extraction'; factor: 1.3897) in order to calculate the final product titers.

Finally, we analyzed if the presence of an *n*-dodecane layer positively or negatively affects the β-caryophyllene formation in *R. capsulatus* cells during cultivation. Therefore, an experiment with repeated sesquiterpene extraction out of production cultures that had been cultivated (3 days) with and without an *n*-dodecane layer before extraction was performed over four days (**Figure S4**). For the analysis of the β-caryophyllene producing *R. capsulatus* cultures grown without a solvent layer, equal amounts of *n*-dodecane were added after cultivation and prior to the extraction procedure.

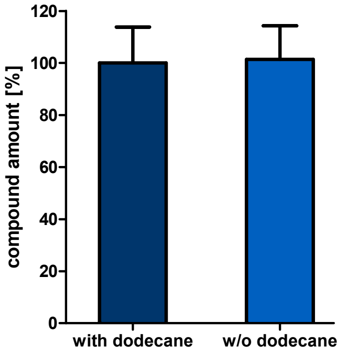

Figure S4. Comparison of relative β-caryophyllene formation in *R. capsulatus* production strains cultivated with (dark blue) and without an *n*-dodecane layer (light blue). Data was normalized to the amount of sesquiterpene extracted from cultures with *n*-dodecane. See text above for details. For *R. capsulatus* cultures containing the *n*-dodecane layer, 500 µL of the solvent was added before cultivation. In contrast, the same amount of solvent was added to cultures without *n*-dodecane after the cultivation. Compounds were extracted and analyzed as described above. Values are means of triplicate measurements. Error bars indicate the respective standard deviations.

Almost no changes of β-caryophyllene formation could be observed in the absence of the *n*-dodecane layer. Hence, the *n*-dodecane layer can alternatively be added after cultivation of the *Rhodobacter* production strains prior to the extraction procedure without any product losses. However, it must be considered that an addition of the organic solvent after cultivation is only reasonable for non-volatile terpenoids, as otherwise the product loss could be excessively high.

In summary, product titers of *R. capsulatus* β-caryophyllene production cultures were determined by analysis of *n*-dodecane extraction samples from disrupted cells. To this end, *R. capsulatus* strains were cultivated without the solvent, disrupted and then extracted one time with *n*-dodecane. Using the calibration curves obtained with reference compounds (**Figure S5**) and taking into account the cultivation parameters v and r_D, as well as the above described results on losses of this procedure (**Figure S2** and **S3**), equation 1 was used for calculating the final β-caryophyllene titers. The dodecane volume ratio r_D represents the ratio of the dodecane volume which was used for the calibration curve (1 mL) to the dodecane volume which was used as a solvent layer during extraction of the production cultures.

$$\text{compound amount}\left[\frac{mg}{L\ culture}\right] = \frac{pa}{s*v*r_D} * 1000\ mL * c_t * c_{ex} \quad (1)$$

$$\boldsymbol{pa = peak\ area\ [pA*s]}$$
$$\boldsymbol{s = slope\ of\ calibration\ curve}$$
$$v = culture\ volume\ [mL]$$
$$r_D = dodecane\ volume\ ratio$$
$$c_t = transfer\ efficiency\ coefficient$$
$$c_{ex} = coefficient\ for\ repeated\ extraction$$

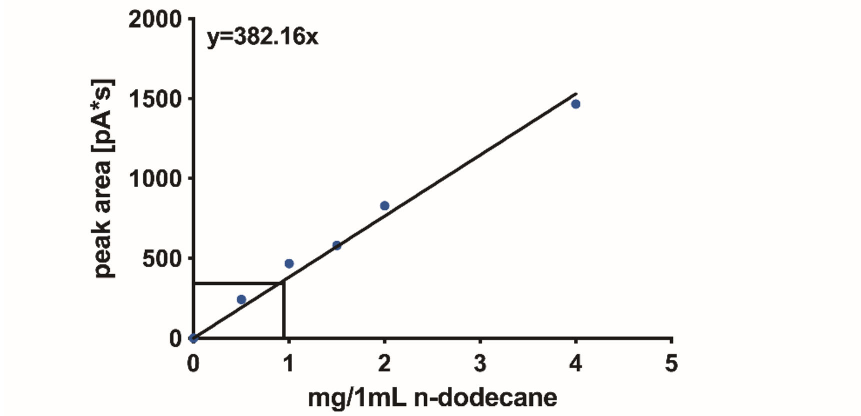

Figure S5. Quantification of extracted β-caryophyllene *via* a calibration curve of β-caryophyllene reference signals in GC-FID analyses. The signal intensities of the authentic reference compound, which were measured as peak areas [pA*s], were correlated to compound quantities [mg] in 1 mL solvent in different concentrations. Mean values of detected signals of *n*-dodecane extraction samples from disrupted cells of the best *R. capsulatus* production strain (SB1003-MVA with pRhon5Hi-2-QHS1-ispA) are indicated (black lines from Y-axis to calibration line). As the extracts from *R. capsulatus* cultures were prepared with *n*-dodecane volumes of 500 µL for hungate cultures and 150 µL for screw neck vials, the extracted amount of biosynthetic product [mg] could be calculated from the linear equations in consideration of the respective solvent amount. These data were used for calculation of product levels in cultures, taking the used culture volume, and factors r_D, c_t and c_{ex} into account, as described above.

2. Supplementary data

Emission range of used light sources and *in vivo* absorption spectrum of phototrophically grown *R. capsulatus* culture

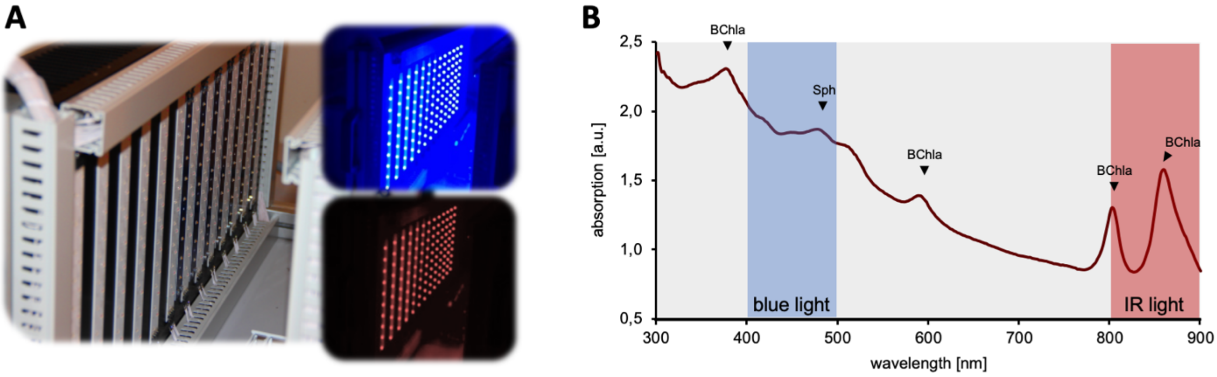

Figure S6. Emission range of different light sources and the absorption spectrum of phototrophically cultivated *R. capsulatus* cells. To illuminate phototrophic cell cultures of *R. capsulatus* more specifically, a custom-made LED panel (**A**) was developed by Vossloh-Schwabe Lighting Solutions GmbH & Co. KG (Kamp-Lintfort, Germany). Each LED array, carries 130 IR (λ_{max}= 850 nm, max. 5.6 mW cm^{-2}) and 130 blue-light LEDs (λ_{max} = 455 nm, 4.0 mW cm^{-2}) on a joint area of 1950 cm^{2} (697 x 280 mm), suitable for specific excitation of photopigments spheroidene (indicated as Sph) and bacteriochlorophyll *a* (BChl *a*). In addition, the array offers 63 UV-A LEDs (λ_{max}= 364 nm, max. 7.6 mW cm^{-2}) in a separate area of 900 cm^{2} (361 × 250 mm), suitable for applying photocaged inducers (see for example [128]) for future optogenetic approaches with *R. capsulatus*. Light intensity quantifications were conducted using a Thermal Power Sensor (S302C, Thorlabs Inc, USA). The emission maxima for the following light sources are shown in (**B**): (i) Blue LEDs emit blue light at around 450 nm (blue area) and (ii) IR LEDs emit light at around 850 nm (light red area). Additionally, a whole-cell absorption spectrum of a *R. capsulatus* culture is shown (dark red line). The three peaks at around 480 nm represent the carotenoid spheroidene, while the peaks at 380 nm, 600 nm, 800 nm and 860 nm represent BChl *a*.

Specifications of cultivation vessels for anaerobic growth of *R. capsulatus* cultures.

Table S2. Cultivation vessel specifications.

Cultivation vessel	Hungate	Screw neck vial
Vessel Specification	Hungate tube (Bellco Glass)	Screw neck vial N13, clear (MN)
Total volume	16.5 mL	4.5 mL
Working volume	15 mL	4.2 mL
Glass type	Type 1 class B borosilicate glass, transmission > 90% at 850 nm	Type 1 borosilicate glass (FIOLAX®), transmission > 90% at 850 nm
Wall thickness	1.05 mm	0.95 mm
Lateral surface area	~ 60.3 cm^{2}	~ 18.5 cm^{2}
Surface-to-volume-ratio	4.02 cm^{-1}	4.40 cm^{-1}

Productivities of β-caryophyllene in *R. capsulatus* SB1003 cultures.

Table S3. Production titers of β-caryophyllene in *R. capsulatus* SB1003 cultures.

**Compound**	**β-caryophyllene**	
Rhodobacter capsulatus strain	SB1003-MVA/pRhon5Hi-2-QHS1-ispA	
Cultivation vessel	Hungate tube	Screw neck vial
Titer at below defined time point (mg L^{-1} culture)	90.39 ± 18.61 mg L^{-1}	139.29 ± 31.35 mg L^{-1}
Time point of highest titer (h)	72 h	72 h
OD at the time point of highest titer (660 nm)	2.79 ± 0.09	2.50 ± 0.06
Cell mass at the time point of highest product titer (gDCW L^{-1} culture)	1.67 ± 0.05 gDCW L^{-1}	1.50 ± 0.04 gDCW L^{-1}
Volumetric productivity (mg L^{-1} h^{-1})[1]	1.26 ± 0.26 mg L^{-1} h^{-1}	1.93 ±0.44 mg L^{-1} h^{-1}
Specific yield at above defined time point (mg $gDCW^{-1}$)	54.01 ± 9.64 mg $gDCW^{-1}$	93.41 ±23.40 mg $gDCW^{-1}$
Specific productivity (mg $gDCW^{-1}$ h^{-1})[1]	0.75 ± 0.13 mg $gDCW^{-1}$ h^{-1}	1.30 ±0.32 mg $gDCW^{-1}$ h^{-1}

[1] Productivities per hour were calculated based on product levels that were present at the time points when highest titers were reached and are thus not necessarily maximal productivities.

Codon optimized DNA sequence of β-caryophyllene synthase QHS1 from *A. annua* for expression in *R. capsulatus*.

The shown sequence encompasses the QHS1 gene referring to the mRNA sequence published in GenBank: AF472361.1, encoding the QHS1 protein (UniProtKB - Q8SA63). At the 5′-end, the synthetic DNA fragment carries a short sequence derived from pET22b (between the *Xba*I and *Nde*I sites) harboring the corresponding ribosome binding site (RBS).

*Xba*I **RBS** Nde*I*/**Start**

TCTAGAAATAATTTTGTTTAACTTTAAG**AAGGAG**ATATACAT**ATG**AGCGTGAAAGAAGAAAAAGTGATC
CGCCCGATCGTGCATTTCCCGCCGAGCGTGTGGGCGGATCAGTTCCTGATCTTCGATGATAAACAGGCGG
AACAGGCGAACGTGGAACAGGTGGTGAACGAACTGCGCGAAGATGTGCGCAAAGATCTGGTGAGCAGC
CTGGATGTGCAGACCGAACATACCAACCTGCTGAAACTGATCGATGCGATCCAGCGCCTGGGCATCGCG
TATCATTTCGAAGAAGAAATCGAACAGGCGCTGCAGCATATCTATGATACCTATGGCGATGATTGGAAA
GGCCGCAGCCCGAGCCTGTGGTTCCGCATCCTGCGCCAGCAGGGCTTCTATGTGAGCTGCGATATCTTCA
AAAACTATAAAAAAGAAGATGGCAGCTTCAAAGAAAGCCTGACCAACGATGTGGAAGGCCTGCTGGAA
CTGTATGAAGCGACCTATCTGCGCGTGCAGGGCGAAGGCGTGCTGGATGATGCGCTGGTGTTCACCCGCA
CCTGCCTGGAAAAAATCGCGAAAGATCTGGTGCATACCAACCCGACCCTGAGCACCTATATCCAGGAAG
CGCTGAAACAGCCGCTGCATAAACGCCTGACCCGCCTGGAAGCGCTGCGCTATATCCCGATGTATGAAC
AGCAGGCGAGCCATAACGAAAGCCTGCTGAAACTGGCGAAACTGGGCTTCAACCTGCTGCAGAGCCTGC
ATCGCAAAGAACTGAGCGAAGTGAGCCGCTGGTGGAAAGGCCTGGATGTGCCGAACAACCTGCCGTAT
GCGCGCGATCGCATGGTGGAATGCTATTTCTGGGCGCTGGGCGTGTATTTCGAACCGAAATATAGCCAGG
CGCGCATCTTCCTGGCGAAAGTGATCAGCCTGGCGACCGTGCTGGATGATACCTATGATGCGTATGGCAC
CTATGAAGAACTGAAAATCTTCACCGAAGCGATCCAGCGCTGGAGCATCACCTGCATCGATATGCTGCC
GGAATATCTGAAACTGCTGTATCAGGGCGTGCTGGATATCTATATCGAAATGGAAGAAATCATGGGCAA
AGAAGGCAAAGCGCATCATCTGAGCTATGCGAAAGAAAGCATGAAAGAGTTCATCCGCAGCTATATGAT
GGAAGCGAAATGGGCGAACGAAGGCTATGTGCCGACCGCGGAAGAACACATGAGCGTGGCGTTCGTGA
GCAGCGGCTATAGCATGCTGGCGACCACCTGCTTCGTGGGCATGGGCGATATCGTGACCGATGAAGCGTT
CAAATGGGCGCTGACCAAACCGCCGATCATCAAAGCGAGCTGCGCGATCGCGCGCCTGATGGATGATAT
CCATAGCCAGAAAGAAGAAAAAGAACGCATCCATGTGGCGAGCAGCGTGGAAAGCTATATGAAACAGT
ATGATGTGACCGAAGAACATGTGCTGAAAGTGTTCAACAAAAAAATCGAAGATGCGTGGAAAGATATC
ACCCGCGAAAGCCTGGTGCGCAAAGATATCCCGATGCCGCTGATGATGCGCGTGATCAACCTGGCGCAG
GTGATGGATGTGCTGTATAAACATAAAGATGGCTTCACCAACGTGGGCGAAGAACTGAAAGATCATATC
AAAAGCCTGCTGGTGCATCCGATCCCGATC**TGA**AAGCTT

Stop *Hind*III

Bioactivities of β-caryophyllene and β-caryophyllene oxide against phytopathogenic fungi

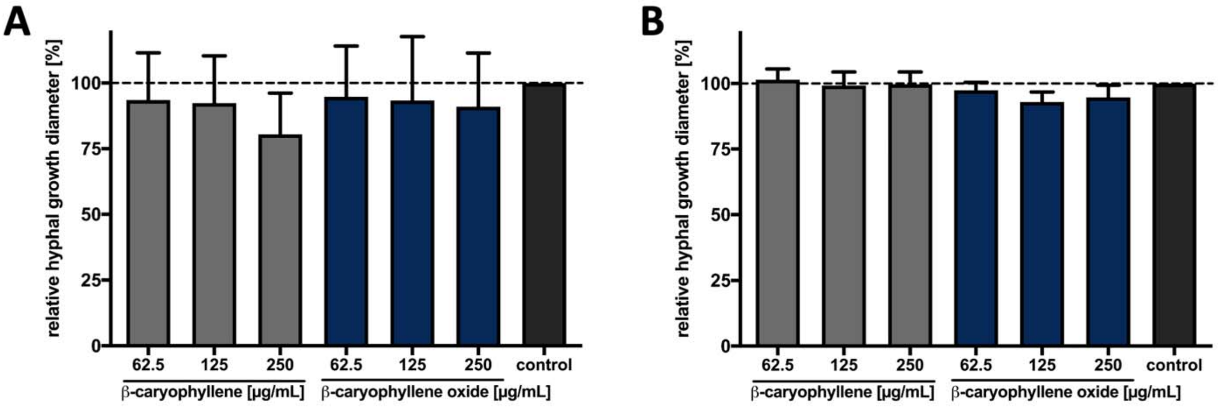

Figure S7. Effect of β-caryophyllene and β-caryophyllene oxide on the hyphal growth of plant pathogenic fungi. Effect of β-caryophyllene and β-caryophyllene oxide against A) *P. lingam* and B) *R. solani*. Final concentration of 62.5 µg mL^{-1}, 125 µg mL^{-1} and µg mL^{-1} of β-caryophyllene (gray bars) and β-caryophyllene oxide (blue bars) in PDA growth medium were used. Medium mixed with the solvents DMSO and Tween 20 (final concentrations, 0.5% and 1% *v*/*v*, respectively) was used as control. Fungal mycelium was placed in the center of each plate and incubated for 7 days at 24 °C. Subsequently, the diameter of the fungal colony was measured, and the relative growth percentage was calculated. Each bar represents the mean ± standard deviation of 3 independent biological replicates ($n = 9$). No significant differences based on ANOVA test ($P < 0.05$).

References

39. Troost, K.; Loeschcke, A.; Hilgers, F.; Özgür, A.Y.; Weber, T.M.; Santiago-Schübel, B.; Svensson, V.; Hage-Hülsmann, J.; Habash, S.S.; Grundler, F.M.W.; et al. Engineered *Rhodobacter capsulatus* as a Phototrophic Platform Organism for the Synthesis of Plant Sesquiterpenoids. *Front. Microbiol.* **2019**, *10*, 1998.

81. Hanahan, D. Studies on transformation of *Escherichia coli* with plasmids. *J. Mol. Biol.* **1983**, *166*, 557–580.

82. Simon, R.; Priefer, U.; Pühler, A. A Broad Host Range Mobilization System for *In Vivo* Genetic Engineering: Transposon Mutagenesis in Gram Negative Bacteria. *Bio/Technology* **1983**, *1*, 784–791.

83. Strnad, H.; Lapidus, A.; Paces, J.; Ulbrich, P.; Vlcek, C.; Paces, V.; Haselkorn, R. Complete genome sequence of the photosynthetic purple nonsulfur bacterium *Rhodobacter capsulatus* SB1003. *J. Bacteriol.* **2010**, *192*, 3545–3546.

88. Rodriguez, S.; Kirby, J.; Denby, C.M.; Keasling, J.D. Production and quantification of sesquiterpenes in *Saccharomyces cerevisiae*, including extraction, detection and quantification of terpene products and key related metabolites. *Nat. Protoc.* **2014**, *9*, 1980–1996.

128 Hogenkamp, F.; Hilgers, F.; Knapp, A.; Klaus, O.; Bier, C.; Binder, D.; Jaeger, K.-E.; Drepper, T.; Pietruszka, J. Effect of Photocaged Isopropyl β-D-1-Thiogalactopyranoside Solubility on Light-Responsiveness of LacI-controlled Expression Systems in Different Bacteria. *ChemBioChem* **2020**, doi:10.1002/cbic.202000377.
